# Reproducibility of antimicrobial test methods

**DOI:** 10.1038/s41598-018-30282-3

**Published:** 2018-08-22

**Authors:** Albert E. Parker, Martin A. Hamilton, Darla M. Goeres

**Affiliations:** 1 Center for Biofilm Engineering, Montana State University, Bozeman, Montana, USA; 2 Department of Mathematical Sciences, Montana State University, Bozeman, Montana, USA

## Abstract

We review reproducibility results for methods that test antimicrobial efficacy against biofilms, spores and bacteria dried onto a surface. Our review, that included test results for *Pseudomonas aeruginosa*, *Salmonella choleraesuis* and *Bacillus subtilis*, suggests that the level of reproducibility depends on the efficacy of the antimicrobial agent being tested for each microbe and microbial environment. To determine the reproducibility of a method, several laboratories must independently test the same antimicrobial agent using the method. Little variability among the efficacy results suggests good reproducibility. Such reproducibility assessments currently are hampered by the absence of an objective process for deciding whether the variability is sufficiently small. We present a quantitative decision process that objectively determines whether any method that assesses antimicrobial efficacy is reproducible. Because the perception of acceptable reproducibility may differ among stakeholders, the decision process is governed by a stakeholder’s specifications that necessarily includes the efficacy of the agents to be tested.

## Introduction

Reproducibility, a cornerstone of the scientific method, is now receiving increased emphasis in the scientific world because too many research findings cannot be reproduced by independent investigators^1,2^. Recently, there was a call for evidence-based evaluations of research methodologies and the development of an associated decision process for determining reproducibility^3^. In response to that call we provide such a decision process that can be applied to any laboratory method.

The reproducibility of any laboratory method can be evaluated using data obtained from a multi-laboratory study. Such reproducibility assessments for antimicrobial test methods customarily have been conducted by standard setting organizations such as ASTM International and AOAC International. However, even in guidance documents published by standard setting organizations, procedures for making judgements about reproducibility are either unavailable or vague^4,5^. Many times these judgements have relied on historical precedent. There is a compelling need for an objective, transparent tool for deciding whether a laboratory method is acceptably reproducible given data from multiple laboratories. This need prompted us to review several multi-laboratory studies, conducted over two decades, of standardized antimicrobial test methods and then to apply the statistical decision process presented here.

Table 1 provides a brief summary of the antimicrobial test methods that we reviewed^6–15^. The methods are categorized by the microbial environment in which the antimicrobial is applied: bacteria dried onto a surface, bacterial spores dried onto a surface, or a bacterial biofilm. For each of the methods, the basic data are viable cell counts recorded as colony forming units (CFU) for microbial preparations that have been treated by an antimicrobial agent and for untreated microbial preparations (i.e., controls). The efficacy outcome of interest from a single application of the method (i.e., a test) is the log reduction (LR), i.e., the reduction of log_10_-transformed CFU counts. For each test condition (microbial species, antimicrobial agent, etc.) in a multi-laboratory study, the main outcomes are the average LR (across laboratories) and the reproducibility standard deviation (SD) (S_R_)^16^. An S_R_ near zero indicates a method exhibiting excellent reproducibility whereas a large S_R_ indicates a method exhibiting poor reproducibility. Deciding whether a test method is reproducible amounts to deciding whether S_R_ is small enough.

**Table 1:** Summary of methods reviewed.

Type	Method	Abb.	Mic.	Org.	Standard	Ref.	Num. Labs	Num. agents	LR Range
dried surface	Hard Surface Carrier Test v1	HSCT1	*P.a*.,	AOAC	991.47	^6^	8	6	6.2–8.2
			*S.c*..			^7^	7	11	6.3–8.5
	Hard Surface Carrier Test v2	HSCT2	*P.a*	AOAC	991.47	^8^	7	11	6.7–8.1
	Use-dilution Method v1	UDM1	*P.a*	AOAC	964.02	^9^	5	2	7.2–8.3
	Use-dilution Method v2	UDM2	*P.a*	AOAC	964.02	^9^	5	2	7.1–8.1
spore	Quantitative Carrier Test	QCT	*B.s*.	ASTM	E2111	^10^	14	16	0–7.9
						[7]	2	3	3.6–7.2
	Sporicidal Activity Test	SAT	*B.s*.	AOAC	966.04	^11^	2	4	5.6–7.2
	Three Step Method	TSM	*B.s*.	ASTM	E2414	^12^	8	9	0–7.5
biofilm	Minimum Biofilm Eradication Concentration	MBEC	*P.a*.	ASTM	E2799	^13^	8	24	−0.2–5.6
	Single Tube Method v1	STM1	*P.a*.	ASTM	E2871	^14^	7	6	2.3–4.6
	Single Tube Method v2	STM2	*P.a*.	EPA	MB-20	^15^	7	4	4.1–8.6

The summary includes the abbreviation (Abb.) used to refer to each method throughout the paper, the microbe (Mic.) tested, the organization (Org.) that standardized the method, and a reference (Ref.) that describes the method and the associated collaborative study in detail. The microbes used with each method are abbreviated as follows: *P.a*. for *Pseudomonas aeruginosa*, *S.c*. for *Salmonella choleraesuis* and *B.s*. for *Bacillus subtilis*.

When developing a new method, investigators initially will strive for good within-laboratory precision; i.e., good repeatability. Repeatability of an antimicrobial test method is quantified by the repeatability SD (S_r_) calculated from the LRs from replicate tests within a single laboratory. Good repeatability is a necessary requirement for good reproducibility (because S_r_ ≤ S_R_). However, repeatability within a laboratory is not sufficient for concluding that the method exhibits acceptable reproducibility among laboratories because, if the LRs greatly vary among the laboratories, S_R_ will be considerably larger than S_r_. Consequently, results from a new antimicrobial test method that has been validated in just one laboratory should be interpreted tentatively.

The decision process that we present for determining acceptable reproducibility (i.e., acceptable values of S_R_) is statistically sound, flexible enough to incorporate different stakeholder specifications, relatively easy to understand, and not dependent on historical precedent. The stakeholder’s reproducibility specifications consist of three quantities which we denote by μ, γ, and δ and will now define. We anticipate that a stakeholder has an application in mind for the antimicrobial agents that are to be evaluated by the method and can specify the ideal true LR value (μ) of the agents for that application. In other words, the stakeholder requires good reproducibility for antimicrobial agents that are expected to produce LRs near μ. The stakeholder will also specify the percentage (γ) of the tests that must produce LRs that differ from μ by no more than a maximum error (δ). We will consider the specifications γ = 90%; δ = 1, 2, or 3; and, depending on the antimicrobial test method, μ between 0 and 9. The specifications, coupled with data from a multi-laboratory study of an antimicrobial test method, determine how small the reproducibility SD, S_R_, must be in order to justifiably conclude that the method is acceptably reproducible. The decision is guided by reference to a graph (presented herein) that clearly shows how acceptable values of S_R_ relate to the specifications, thereby providing a practical tool for reproducibility judgments. The process is applicable to any antimicrobial test method that produces a quantitative measure of efficacy such as a log reduction that approximately follows a normal distribution.

## Results

Brief descriptions of the antimicrobial test methods that we reviewed and the associated multi-laboratory studies are displayed in Table 1. The studies were not designed to assess any specific antimicrobial agent(s) but rather to evaluate the methods. Commonly used liquid antimicrobial agents were used in the multi-laboratory evaluations. The agents were possibly diluted or concentrated to create a desired range of LRs. The number of tested agent-by-concentration treatments ranged from 2 to 24 across the studies. The reviewed methods tested agents against *Pseudomonas aeruginosa* and *Salmonella choleraesuis* dried onto a surface, *Bacillus subtilis* spores, and *Pseudomonas aeruginosa* biofilms. The number of laboratories that participated in the evaluations of each of the test methods ranged from 2 to 14.

**Figure 1:**
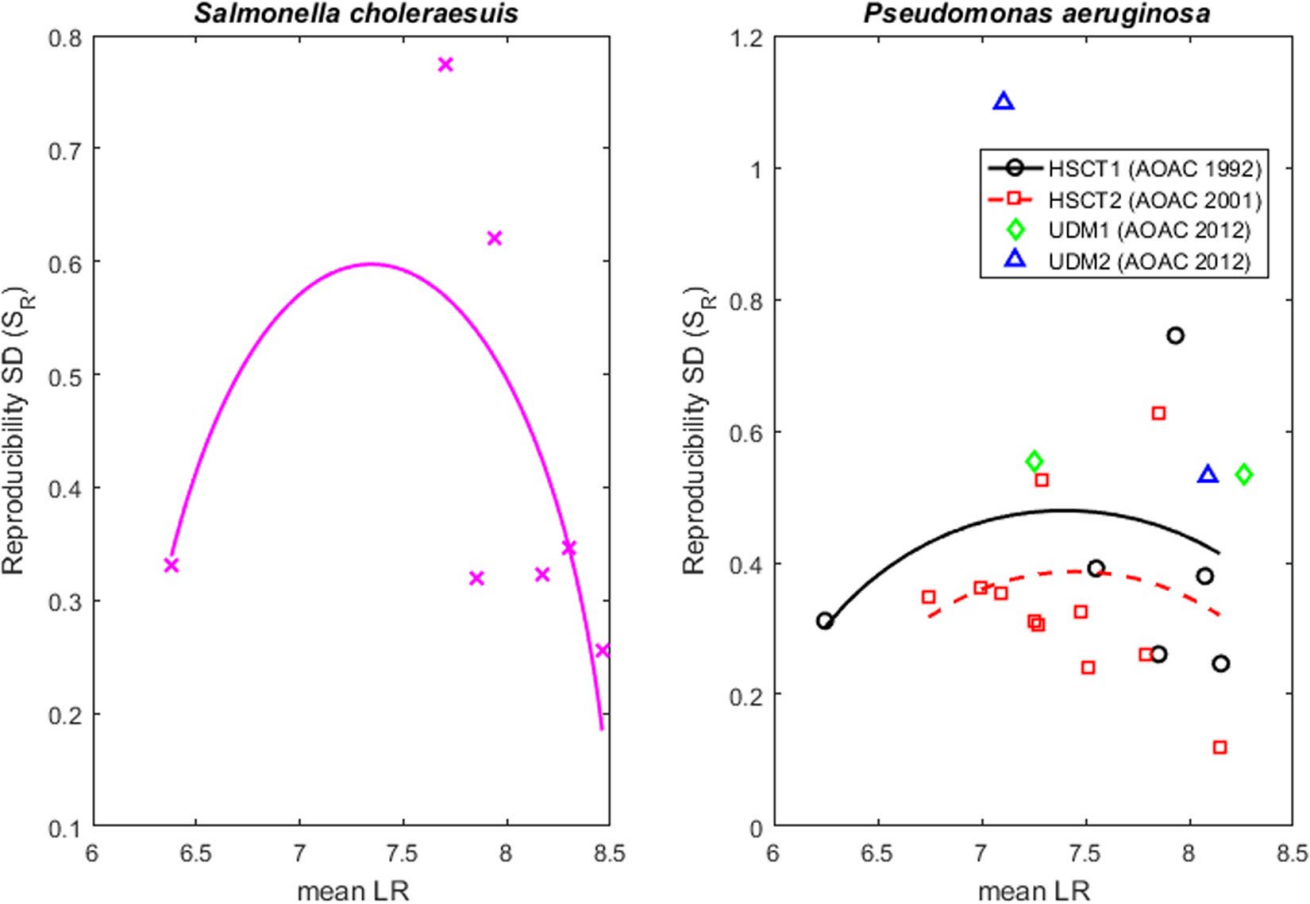
Reproducibility of methods that test antimicrobials against bacteria dried onto a surface. The left pane shows results from a 7-lab study of the HSCT1 that tested agents for efficacy against *S. choleraesuis* dried onto a surface. The right panel shows results from a 8-lab study of the HSCT1, a 7-lab study of the HSCT2, and a 5-lab study of the UDM1 and UDM2 that tested products for efficacy against *P. aeruginosa* dried onto a surface. In both panes, each point corresponds to the reproducibility SD (S_R_) and mean LR attained by a single product in the multi-laboratory study. The regression curves approximate S_R_ as a frown-shaped function of the mean LR for the HSCT1 and HSCT2. It is not possible to fit a regression curve to data from the UDM1 or UDM2 because only two agents were tested in the associated multi-laboratory studies.

**Figure 2:**
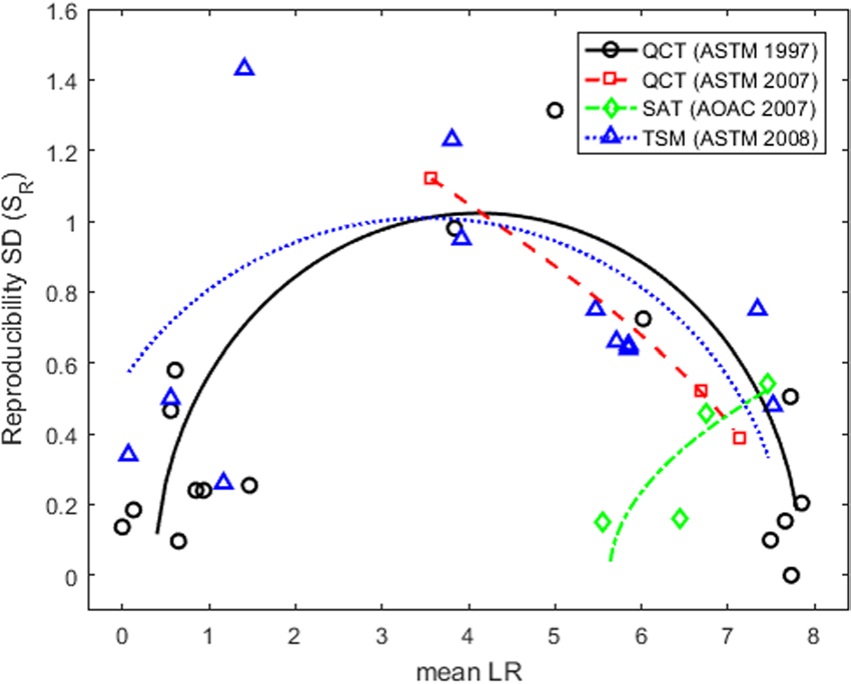
Reproducibility of methods that test sporicides. Results are shown from multi-laboratory studies of 3 different methods that test agents against *B. subtilis* spores. The number of labs and products tested in each study are given in Table 1. The regression curves approximate the reproducibility SD (S_R_) as a frown-shaped function of the mean LR for each method. Each point corresponds to S_R_ and the mean LR attained by a single agent in a multi-laboratory study.

**Figure 3:**
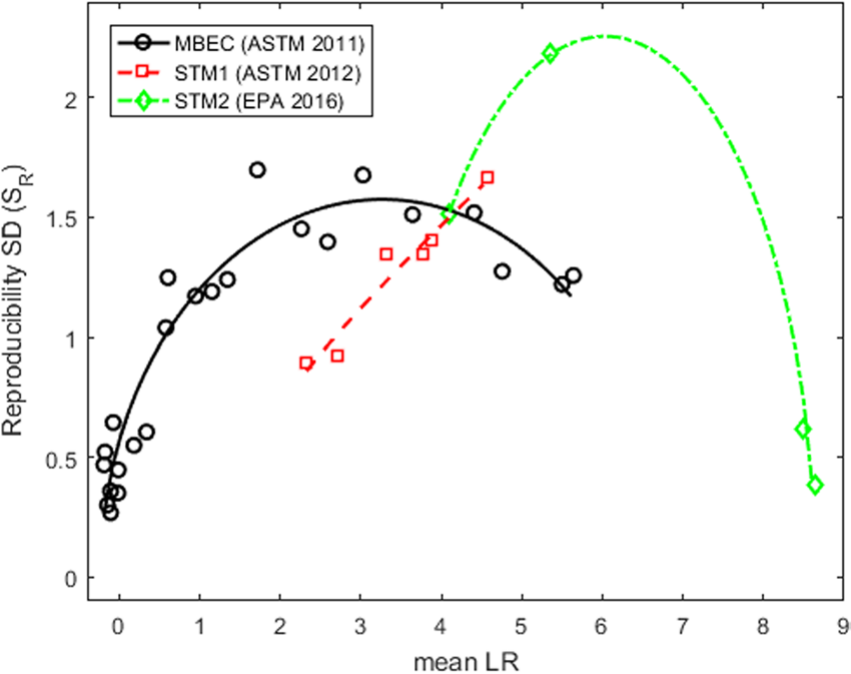
Reproducibility of methods that test antimicrobials against biofilms. Results are shown from multi-laboratory studies of 3 different methods that test agents against *P. aeruginosa* biofilms. The number of labs and products tested in each study are given in Table 1. The regression curves approximate the reproducibility SD (S_R_) as a frown-shaped function of the mean LR for each method. Each point corresponds to S_R_ and the mean LR attained by a single agent in a multi-laboratory study.

Each observed S_R_ was plotted against the associated average LR for each antimicrobial agent, with a separate plot for each microbial environment (Figs 1–3). Regardless of the microbial species or environment, the results when testing ineffective agents and highly effective agents are always more reproducible than the results when testing moderately effective agents. For each test method, the points form a frown-shaped pattern that can be well-approximated by a regression curve (i.e., a quadratic fit to the S_R_^2^ by least squares). The figures show the (square-root transformed) curves that predict S_R_ as a function of the mean LR. This dependence of reproducibility on agent efficacy is why the assessment of reproducibility of an antimicrobial test method must depend on the average LR (µ) of the agent(s) being tested.

Our decision process relies on the calculation of the maximum acceptable S_R_ (S_R,max_) for a method. A method is said to exhibit acceptable reproducibility if and only if the reproducibility SD estimated from a collaborative study satisfies S_R_ ≤ S_R,max_. The value of S_R,max_ depends on the stakeholder specifications (μ, γ, and δ) and two critical inputs from the multi-laboratory study of the test method: the number of laboratories (*I*) and the fraction of the reproducibility variance that is attributable to within-laboratory sources (*F*), *F* = (S_r_/S_R_)^2^, so 0 ≤ *F* ≤ 1. The calculation also depends on the number of replicate tests in each laboratory (*J*), but the decision process is much less sensitive to this aspect of the study design. To illustrate how to calculate *F*, Figs S1 and S2 in the Supplementary Material display the observed S_r_^2^ values against the average LR concurrently with S_R_^2^ for three methods. Just as for S_R_, a consistent frown-shaped relationship is evident for the within-laboratory repeatability SD, S_r_. The ratio of the frown-shaped curves in Figs S1 and S2 is *F*(μ) = (S_r_/S_R_)^2^ which, like S_r_ and S_R_, is a function of the mean LR μ. In other words, the stakeholder’s choice of μ drives the reproducibility acceptability criteria, S_R,max_, via *F*(μ).

**Figure 4:**
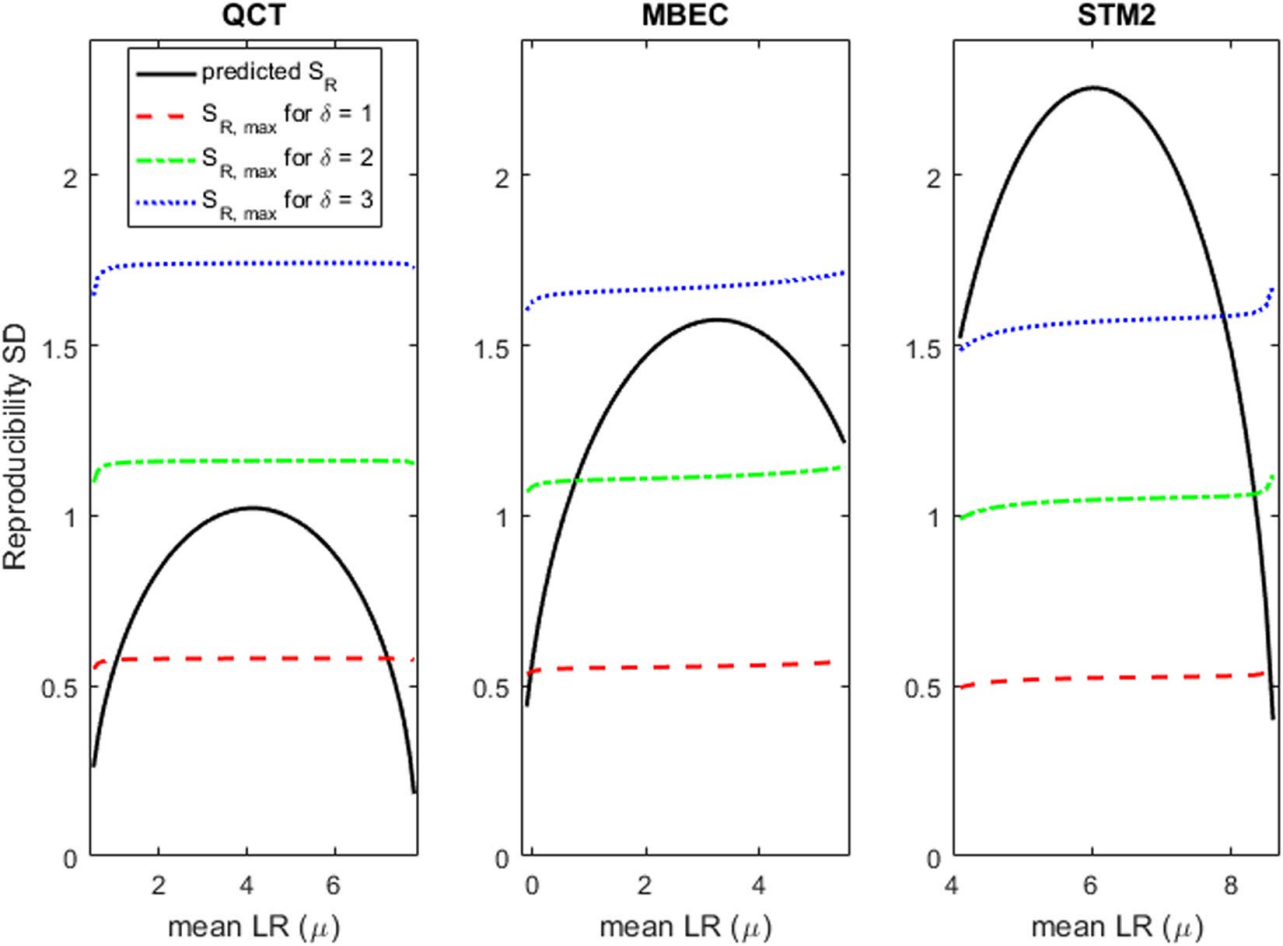
Assessing reproducibility of 3 antimicrobial test methods according to a stakeholder’s specifications. For a range of stakeholder specifications (δ = 1, 2 and 3; 0 < µ < 9; and γ = 90%) reproducibility assessments are provided for 3 of the methods in Table 1. The black curves that show the predicted reproducibility SDs (S_R_) are from Figs 2,3. The curves depicting the maximum acceptable S_R, max_ were calculated from equation (1).

Figure 4 illustrates the decision process applied to three antimicrobial test methods: the QCT sporicide test, and the MBEC and STM2 biofilm tests. For each method the frown-shaped relationship between S_R_ and the mean LR from the multi-laboratory study (from Figs 2–3) is shown. The maximum acceptable SD, S_R,max_, is shown as a function of the stakeholder specifications: γ = 90%, δ = 1, 2, or 3, and 0 ≤ µ ≤ 9. To further illustrate the decision process, consider the QCT, and suppose a stakeholder’s target mean LR is μ = 3, a common target for household liquid antimicrobials. The frown-shaped black curve shows the predicted S_R_ values from Fig. 2; the point on the curve corresponding to μ = 3 is nearly S_R_ = 1. If the stakeholder also specified γ = 90% and δ = 1 then the red dashed line in the QCT panel in Fig. 4 shows that the resulting S_R,max_ value is smaller than S_R_ = 1 at μ = 3. Therefore, for the specifications μ = 3, γ = 90%, and δ = 1, the method is not sufficiently reproducible for the stakeholder’s purposes. However, if the stakeholder had specified δ = 2 then the green dot-dashed line shows that the resulting S_R,max_ is larger than S_R_ = 1 at μ = 3, so the method is sufficiently reproducible for the stakeholder specifications μ = 3, γ = 90%, and δ = 2. The equations in the Methods can be used to show that when δ = 1.7, then S_R,max_ = S_R_ = 1 at μ = 3 indicating that the method is sufficiently reproducible if and only if the stakeholder’s δ is 1.7 or larger.

The panels in Fig. 4 also vividly demonstrate the set of mean LRs, μ, for which the antimicrobial test method exhibits acceptable reproducibility given values of γ and δ. For γ = 90%, each method in Fig. 4 exhibits acceptable reproducibility for any μ where the corresponding position of the frown-shaped solid black curve is below the S_R,max_ curve for the selected δ. For the MBEC results in the second pane of Fig. 4, if a stakeholder requires γ = 90% and δ = 1, the MBEC method is not sufficiently reproducible for any value of μ, and thus not for any antimicrobial agent. For δ = 2, the MBEC method is sufficiently reproducible only when testing agents with a mean LR μ ≤ 0.8. For δ = 3, the MBEC is acceptably reproducible when testing any agent of any efficacy level. Supplementary Table S1 summarizes decisions regarding reproducibility for the QCT, MBEC and STM2.

**Figure 5:**
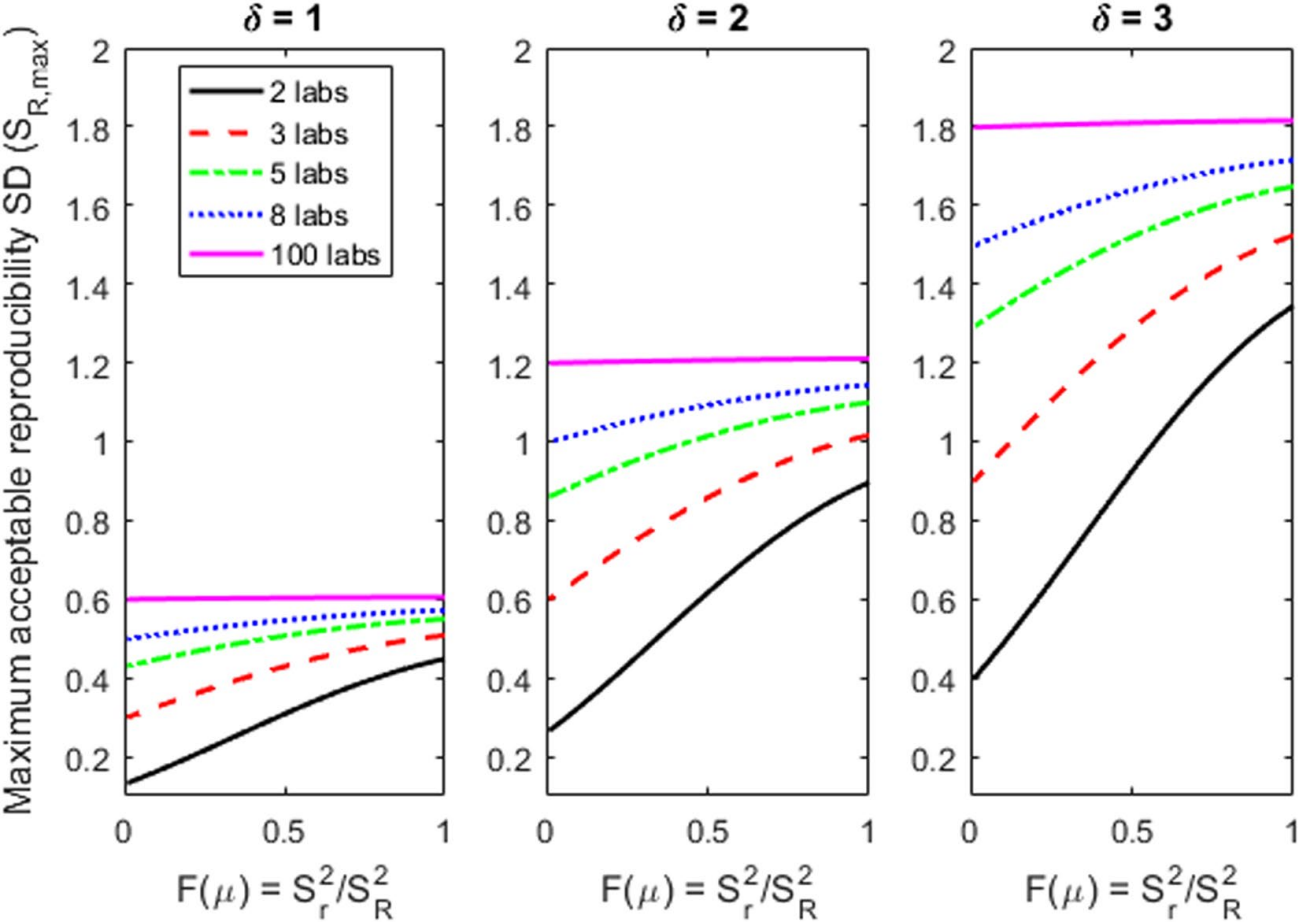
Assessing reproducibility of ANY antimicrobial test method according to a stakeholder’s specifications. For a range of stakeholder specifications (δ = 1, 2 and 3; γ = 90% and *F*(µ) = S_r_^2^/S_R_^2^), the maximum acceptable reproducibility SD of a method is determined, S_R,max_ (via equation (1)). There is 1 other input: the number of labs in the multi-laboratory study of a method.

Figure 5 shows S_R,max_ values for some combinations of *I* (the number of labs) and *F* (the proportion of within-laboratory variance) that could reasonably occur in a multi-laboratory study of an antimicrobial test method when the specifications are γ = 90% and δ = 1, 2 or 3. The figure shows that *F* has little influence on S_R,max_ for large studies (*I* > 14). Figure 5 is a visual tool for assessing the reproducibility of any laboratory method that is relevant for application by most stakeholders. It shows at a glance which specifications, among a spectrum of specifications for δ, produce a decision of acceptable reproducibility of a method. For example, we used the results in Fig. 5 to generate Fig. 4. We advocate this visual tool for determining whether an antimicrobial test method is acceptably reproducible based on the results of a multi-laboratory study. The study team would do the calculations to calculate *F* and *S*_R_ as a function of µ (perhaps using quadratic regression on S_R_^2^as we do here); a stakeholder could then specify γ = 90% and δ of 1, 2 or 3 and then simply read Fig. 5 to determine whether the test method is sufficiently reproducible for the application at hand. For different specifications for γ and/or δ, a figure similar to Fig. 5 would need to be constructed using the simple equations specified in the Methods.

## Discussion

We reviewed antimicrobial test methods standardized by ASTM, AOAC or EPA that have been evaluated by multi-laboratory studies over the past 25 years (Table 1) for which we had access to the complete data, a necessary requirement for applying the same calculations to each study and viewing the results through the same lens. Many of these methods have been considered for regulatory use in the US. The STM2 was recently adopted by EPA as the first method to substantiate biofilm efficacy claims in the US^15^. The SAT is used for sporicide registration^11^. The UDM1 has been used for decades by regulators for testing liquid antimicrobials on nonporous inanimate surfaces, and consequently may be the most used method in our review; hence the results for UDM1 provide a historical benchmark for other antimicrobial test methods. UDM1 was replaced by UDM2 in 2012 as the regulatory workhorse for testing hard surface antimicrobials^9^. The HSCT methods are an alternative to the UDM2 that have been used sparingly by the regulated community^9^. A modified version of the QCT method is currently under study as a possible replacement to the UDM2 for regulatory purposes in the US and Europe^17^.

Manufacturers, regulators and end-users of antimicrobials need to decide which antimicrobial agents work best for different environments and which agents to allow in the marketplace. Consistently (i.e., reproducibly) making correct decisions, thereby positively affecting human health, is possible if a reproducible method is used to test the efficacy of antimicrobial agents. In other words, stakeholders demand methods that are demonstrably reproducible. Our contribution is not to suggest which method to use – stakeholders should choose a laboratory method based on the microbial environment being modeled - but rather to provide an objective process for determining whether a method is sufficiently reproducible for a stakeholder’s application.

For example, we have illustrated that an important element to the decision making process is that an antimicrobial test method’s reproducibility may be deemed acceptable for only a narrow range of LRs. Whether that range of LRs is appropriate depends on the target mean LR, μ, imposed by a stakeholder, such as an industry or regulatory authority, and the environment where the antimicrobial agent will be applied. Consider the results for QCT and STM2. Because QCT is acceptably reproducible to ensure that γ = 90% of tests generate LRs within δ = 2 of the true mean LR (Fig. 4), if QCT were used to test agents against a required LR of 3 (this is the requirement for liquid antimicrobial agents on non-food contact surfaces in the US^18^), then manufacturers of antimicrobials would need to produce agents that achieve a target mean LR of 5 ( = 3 + δ) in order to pass at least 95% of QCT tests. Tests of antimicrobials against biofilms using the STM2 require LRs ≥ 6^15^. Because the STM2 generates γ = 90% of tests with LRs within δ = 2 of the true mean LR when the true mean LR is greater than 8.3, then manufacturers would need to produce agents that achieve a target mean LR of 8.3 to pass at least 95% of STM2 tests.

Our analysis shows that the dried surface methods exhibit excellent reproducibility compared to the other methods reviewed, whereas the biofilm methods exhibit the least level of reproducibility. Although these results pertain only to the methods in Table 1 for *P. aeruginosa*, *S. choleraesuis* and *B. subtilis*, they suggest that, in addition to being more resistant to antimicrobials (e.g., see^19–21^), bacterial biofilms are also more variable when responding to antimicrobials compared to either spores or bacteria dried onto a surface.

The smooth, frown-shaped relationship between the reproducibility SD, S_R_, and the average LR was a consistent feature of the multi-laboratory studies that we reviewed regardless of the microbial species or environment (Figs 1–3). The frown-shaped dependence of S_R_ on the average LR has been pointed out previously^20,22,23^. Fortunately, for the methods that we reviewed, the relationship between reproducibility and efficacy is smooth enough to be described by a simple regression curve. Although a different antimicrobial test method may display a different pattern of S_R_ values, some interpolation between the few reproducibility results generated by a multi-laboratory study is necessary to estimate S_R_ as a continuous function of the average LR. This continuous curve allows one to predict a method’s S_R_ for any target mean LR, μ, specified by a stakeholder. A similar curve can also be generated (Figs S1 and S2) to predict a method’s repeatability S_r_ across tests within a single laboratory for any µ. These curves for S_r_ and S_R_ are also a prerequisite for applying the decision tool, namely the calculation of the maximum acceptable reproducibility SD (S_R,max_) for any μ specified by a stakeholder.

Judging the reproducibility of an antimicrobial test method amounts to deciding whether the observed S_R_ at a given level of efficacy, μ, is small enough. That judgment has been problematic because until now statistical decision criteria have not been established for the reproducibility S_R_ for an antimicrobial test method. Tilt and Hamilton^24^ suggested a reproducibility acceptance criterion of S_R_ ≤ 1.5 based on a review of suspension and dried surface tests of commonly-used agents against the same laboratory microbes considered here (as well as others). Our results suggest that using a single value such as S_R, max_ = 1.5 can be too simplistic.

Instead, Figs 4 and 5 show that the maximum allowable reproducibility SD (S_R,max_) is a non-constant, non-linear function of µ, *F* and *I*. The non-linearity is because it is more challenging to reproducibly generate LRs when the method’s variability is dominated by among-laboratory sources (i.e., S_R,max_ is lower when *F* is small). For a large study (*I* > 14 laboratories), Fig. 5 confirms the statistical theory that S_R,max_ is approximately a constant δ/1.645 when γ = 90% regardless of µ or *F*, in which case, the Tilt and Hamilton acceptance criterion of S_R_ ≤ 1.5 corresponds to the stakeholder specification δ = 2.5 when γ = 90%.

The methods that we reviewed (Table 1) quantified antimicrobial efficacy as a LR based on plate count data (CFUs). Not surprisingly, other methods might quantify efficacy differently, e.g. via bio-volumes estimated from confocal microscopy^25^ or via amplification of DNA by PCR or qPCR^26^. Our statistical approach for assessing the reproducibility of an antimicrobial test method can be applied regardless of how efficacy is quantified. However, the approach would be simplified if the variability of the efficacy response is not dependent on the level of efficacy as we have shown is the case for LRs based on CFUs (Figs 1–3).

The decision process will be informative to those who design multi-laboratory studies. A display such as Fig. 5 can be calculated for a set of possible multi-laboratory designs, thereby previewing the range of potential S_R,max_ values. Before initiating the multi-laboratory study, an antimicrobial test method usually is thoroughly evaluated by a single laboratory. Such an evaluation produces a repeatability SD, S_r_, for a range of average LR outcomes. If the *a priori* calculations of S_R,max_ for reasonable assumed values for *F* and a potential study design (with *I* laboratories and *J* tests at each laboratory) show that the frown-shaped curve for S_r_ is too high, then S_R_ must also be too high and the multi-laboratory study should be reconsidered, perhaps shelved. On the other hand, if S_r_ is small enough to justify a multi-laboratory study, the *a priori* S_R,max_ calculations can guide the study design (i.e., how many labs to include, and how many experiments to be conducted by each lab).

The reproducibility decision process that we present can be adapted to assess any quantitative laboratory method that has been evaluated by a multi-laboratory study. The decision process for antimicrobial test methods depends on the average LR of the antimicrobial agents being tested because, as we have shown, the reproducibility of these methods depends on the efficacy of the agents. The process is simpler when assessing the reproducibility of laboratory methods for which S_R_ does not depend on the expected method outcome.

## Materials and Methods

Detailed descriptions for the reviewed test methods are provided in the citations listed in Table 1. The LRs for each agent in each multi-laboratory study were analyzed by a linear mixed effects model using the method of restricted maximum likelihood^16,27,28^. Each analysis provided the repeatability variance (S_r_^2^), the among-laboratory variance (S_lab_^2^) and the associated mean LR for the method. The reproducibility SD was then calculated by S_R_ = [S_r_^2^ + S_lab_^2^]^½^.

Conventional diagnostic checks were performed to assess model fit^16^. For example, we used residual plots to investigate potential outliers, confirm the homogeneous variance assumption, and to assess that the residuals approximately followed a normal distribution.

These calculations, notation, and terminology are consistent with guidelines published by ASTM^4^ and AOAC^5^, sources that discuss only balanced multi-laboratory studies, that is, studies in which each laboratory conducts *J* tests of the agent. Calculations were performed using the software R^29^ package *nlme*^30^. Explicit R code used for the analysis of a multi-laboratory data set is available on-line^31^.

For each antimicrobial test method, a quadratic regression model was fit separately to the variances S_R_^2^’s and the S_r_^2^’s as a function of the mean LR. The variances were directly modeled as opposed to modeling the SDs because S_R_^2^ is an unbiased estimator of the true reproducibility variance (σ_R_^2^) whereas S_R_ is a biased estimator of σ_R_. This means that a normal distribution was used to approximate the scaled chi-square distribution of the residuals from the quadratic model. Gamma and weighted normal quadratic models (S_R_^2^′s weighted by their degrees of freedom) were also investigated, but these are not presented here.

We derived equations for calculating S_R,max_ by adapting a statistical technique advocated by pharmaceutical statisticians for assessing the reproducibility of chemical assay methods^32–35^. The computations are based in turn on a procedure for calculating a γ-expectation tolerance interval^36^ for a LR from an individual test, $\overline{{\rm{LR}}}\pm T(I,J,F)\times {S}_{{\rm{R}}}$ where the *t*-multiplier *T*(*I, J, F*) is defined below. The following shows how to calculate S_R,max_ given the stakeholder specifications μ,δ and γ and the characteristics of the multi-laboratory study (the number of laboratories (*I*), and the number of tests conducted at each laboratory (*J*)):

1 $${{\rm{S}}}_{{\rm{R}},\max }=\delta /T(I,J,F(\mu ));$$

2 $$T(I,J,F(\mu ))={t}_{(1-{\rm{\gamma }}/100)/2,df}\sqrt{1+U}.$$

In Equation (2), $U=\frac{{(H/I+1/IJ)}^{2}}{H+1}$, *H* = (S_R_^2^ − *S*_r_^2^)/*S*_r_^2^ = 1/*F* − 1, and ${t}_{(1-{\rm{\gamma }}/100)/2,df}$ is the 1 − (1 − γ/100)/2 quantile from a *t*-distribution having the degrees of freedom approximated by Satterthwaite’s formula,

$$df=\frac{{(H+1)}^{2}}{\frac{{(H+\frac{1}{J})}^{2}}{I-1}+\frac{{(1-\frac{1}{J})}^{2}}{IJ{(1-\frac{1}{J})}^{2}}}.$$

Unlike the expectation tolerance interval derived in^36^, because S_r_ and S_R_ are functions of the mean LR,µ, here we consider *F* as a function of µ. To calculate *F*(µ) = *S*_r_^2^/S_R_^2^ for any µ, we interpolated the S_r_ and S_R_ values using the parabolic regression curves (see Figs S1 and S2).

## Electronic supplementary material

Supplementary Material
