# Supplementary material for: Reproducibility of antimicrobial test methods
Source: Sci Rep. 2018 Aug 22;8:12531. doi: 10.1038/s41598-018-30282-3 (PMC6105646; doi:10.1038/s41598-018-30282-3)
Supplement: Supplementary file 1 — Supplementary Material [file 41598_2018_30282_MOESM1_ESM.docx]

**Supplementary Material:**

**Reproducibility of antimicrobial test methods**

**Albert E. Parker^1,2,*^, Martin A. Hamilton^1,2^, Darla M. Goeres^1^**

^1^Center for Biofilm Engineering, Montana State University, Bozeman, Montana, USA; ^2^Department of Mathematical Sciences, Montana State University, Bozeman, Montana, USA

^*^Correspondence to: Albert E. Parker, [parker@math.montana.edu](mailto:albert.parker@montana.edu)

**Figure S1. Repeatability and Reproducibility of QCT method that tests sporicides.** Each point corresponds to the variance and the mean LR attained by a single agent in the 14-laboratory study of the QCT. The curves show the parabolas fit to the variances. The ratio of the bottom parabola to the top parabola is *F*(µ), the proportion of variance due to within-laboratory sources.

**Figure S2: Repeatability and reproducibility of methods that test antimicrobials against biofilms.** Each point corresponds to the variance and the mean LR attained by a single agent in a multi-laboratory study. The curves show the parabolas fit to the variances. The ratio of the bottom parabola to the top parabola is *F*(µ), the proportion of variance due to within-laboratory sources.

**Table S1: Acceptable reproducibility.** A summary of the decisions regarding reproducibility of the 3 methods in Figure 4 when a stakeholder specifies that δ=90% of the LRs must be within δ = 1, 2 or 3 of the true mean LR. The most restrictive (i.e., smallest) S_R,max_ provides a simple constant value against which to compare S_R_. The range of mean LRs is provided for which a method is acceptably reproducible.

| **Method** | **Mic.** | **Num. Labs** | **Most restrictive acceptable reproducibility SD (S_R, max_)** | | | **Mean LRs that produce acceptable reproducibility**  **(µ≤ or µ≥)** | | |
| --- | --- | --- | --- | --- | --- | --- | --- | --- |
|  |  |  | **δ = 1** | **δ = 2** | **δ = 3** | **δ = 1** | **δ = 2** | **δ = 3** |
| UDM2 | *P.a.* | 5 | 0.54 | 1.08 | 1.63 | ≥8 | ≥8 | ≥8 |
| QCT | *B.s.* | 14 | 0.55 | 1.10 | 1.65 | ≤1 and ≥7.2 | ALL | ALL |
| MBEC | *P.a.* | 8 | 0.53 | 1.07 | 1.60 | NONE | ≤0.8 | ALL |
| STM2 | *P.a.* | 7 | 0.50 | 0.99 | 1.49 | ≥8.6 | ≥8.3 | ≥7.9 |
